# The promotion of cooperation by the poor in dynamic chicken games

**DOI:** 10.1038/srep43377

**Published:** 2017-02-24

**Authors:** Hiromu Ito, Yuki Katsumata, Eisuke Hasegawa, Jin Yoshimura

**Affiliations:** 1 Department of International Health, Institute of Tropical Medicine, Nagasaki University, Nagasaki, 852-8523, Japan; 2 Graduate School of Science and Technology, Shizuoka University, Hamamatsu, 432-8561, Japan; 3 Department of Mathematical and Systems Engineering, Shizuoka University, Hamamatsu, 432-8561, Japan; 4 Laboratory of Animal Ecology, Department of Ecology and Systematics, Graduate School of Agriculture, Hokkaido University, Sapporo 060-8589, Japan; 5 Department of Environmental and Forest Biology, State University of New York College of Environmental Science and Forestry, Syracuse, NY 13210, USA; 6 Marine Biosystems Research Center, Chiba University, Uchiura, Kamogawa, Chiba 299-5502, Japan

## Abstract

The evolution of cooperative behavior is one of the most important issues in game theory. Previous studies have shown that cooperation can evolve only under highly limited conditions, and various modifications have been introduced to games to explain the evolution of cooperation. Recently, a utility function basic to game theory was shown to be dependent on current wealth as a conditional (state) variable in a dynamic version of utility theory. Here, we introduce this dynamic utility function to several games. Under certain conditions, poor players exhibit cooperative behavior in two types of chicken games (the hawk-dove game and the snowdrift game) but not in the prisoner’s dilemma game and the stag hunt game. This result indicates that cooperation can be exhibited by the poor in some chicken games. Thus, the evolution of cooperation may not be as limited as has been suggested in previous studies.

Game theory has been applied to biology to solve questions of cooperative behavior in animals and humans^1^,^2^,^3^,^4^,^5^,^6^,^7^. In humans and certain animals, individuals sometimes risk their lives to help others, e.g., people diving to help drowned children and alarm calls in animals. Why do animals and humans sometimes perform altruistic or cooperative behaviors even if the expected rewards are minimal (or absent) compared with the cost (death)?

When a group (society) consists of closely related individuals, kin selection may explain such altruistic and cooperative behaviors^8^,^9^. However, altruistic and cooperative behaviors often occur between unrelated individuals. For example, people who rescue drowning children are often strangers, and alarm calls help animals of species different from the caller. Present-day human societies are formed primarily by unrelated (non-kin) individuals. In some animal societies, helpers are often not related to their receivers. Thus, the evolution of altruism and cooperation among non-kin members is an important issue in game theory^4^.

Because the evolution of altruism and/or cooperation is highly limited under simple game-theoretic conditions, various modifications have been introduced, e.g., spatial structures^10^,^11^,^12^,^13^,^14^,^15^,^16^,^17^,^18^, population structures^19^,^20^, networks^21^,^22^,^23^, spatial heterogeneity^24^,^25^,^26^, and temporal stochasticity^27^. These models are very successful in showing the evolution of cooperative behavior, but it is uncertain whether societal cooperation indeed evolved under these conditions, as these models are highly complex and sophisticated, and cooperative behavior seems to occur more universally in nature.

The development of game theory was based on the axiomatic system of expected utility theory^1^. The axiomatic system of expected utility theory has recently been found to be a static model based on the comparison of preferences^28^. Because game theory was developed based on this axiomatic system, game theory itself is a static model as well. Therefore, any dynamic extension of game theory should be a quasi-dynamic model that has no objective function (an optimization criterion). Bellman built the first dynamic optimization model, referred to as dynamic programming^29^. Dynamic programming is a numerical algorithm of dynamic optimization based on the “Principle of Optimality.” A theory of dynamic optimization has recently been developed, in which this optimality principle is applied to a stochastic process^30^,^31^. In the development of this theory, the dynamic utility function was derived as a form of maximization of the expected logarithmic growth rate. In a previous study, we applied the dynamic utility theory to the payoff matrix of a hawk-dove game. We specifically considered the interpretation of equal division in a dove-dove contest and showed that the payoffs in the matrix should be interpreted as the amount of gain, instead of utility^28^. In the present study, we apply this interpretation to various games and compare the optimal strategies with those of traditional game theory. Here, we analyze the optimal strategies of two players with various levels of current wealth in a single trial game.

## Theoretical Rationale

First, we explain the derivation of the dynamic utility function^30^,^31^. Let time *t* = 0, …, *T* (final time) and suppose that *w*_*t*_ and *r*_*t*_ are wealth at *t* and the growth rate at *t*, respectively. Note that *r*_*t*_ (>0) is the non-negative state variable of a decision maker (independent, identically distributed random variables). We immediately get *w*_*t*+1_ *=* *r*_*t*_*w*_*t*_. Wealth at the final timestep, *w*_*T*_, is then expressed as follows:


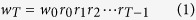


The decision maker can optimize this stochastic process by choosing the best option at every time point in Eq. (1). We therefore maximize the final wealth at *T, w*_*T*_, as follows:


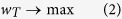


The maximization of *w*_*T*_ (Eq. (2)) is equivalent to that of the geometric mean growth rates, as follows:


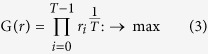


Taking the logarithm of Eq. (3), we obtain the following equation:


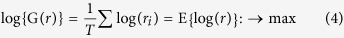


Therefore, we can define the dynamic utility function *u*(*r*) for this maximization:


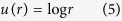


We now maximize the expected dynamic utility E{*u*} = E{log *r*}^32^. From the temporal equation *w*_*t*+1_ = *r*_*t*_*w*_*t*_, we obtain the following equation:


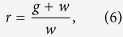


where *g* and *w* are the current gain and the current wealth, respectively. The dynamic utility function (Eq. (5)) is then translated into the function of gain, *g* (decision variable), given current wealth *w* (state variable), as follows:


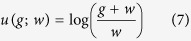


We now maximize the expected utility, E{*u*(*g*; *w*)}, which indicates that current wealth is the state variable.

Thus, the derived dynamic utility is in the form of a logarithmic function (Eq. (7)). Note that the value of *g* satisfies −*w* < *g*. This analytical solution demonstrates that the utility function depends on the current gain and the current wealth status at the time of decision-making. This dynamic utility function is applicable to animals as well as human beings^30^,^31^.

**Figure 1:**
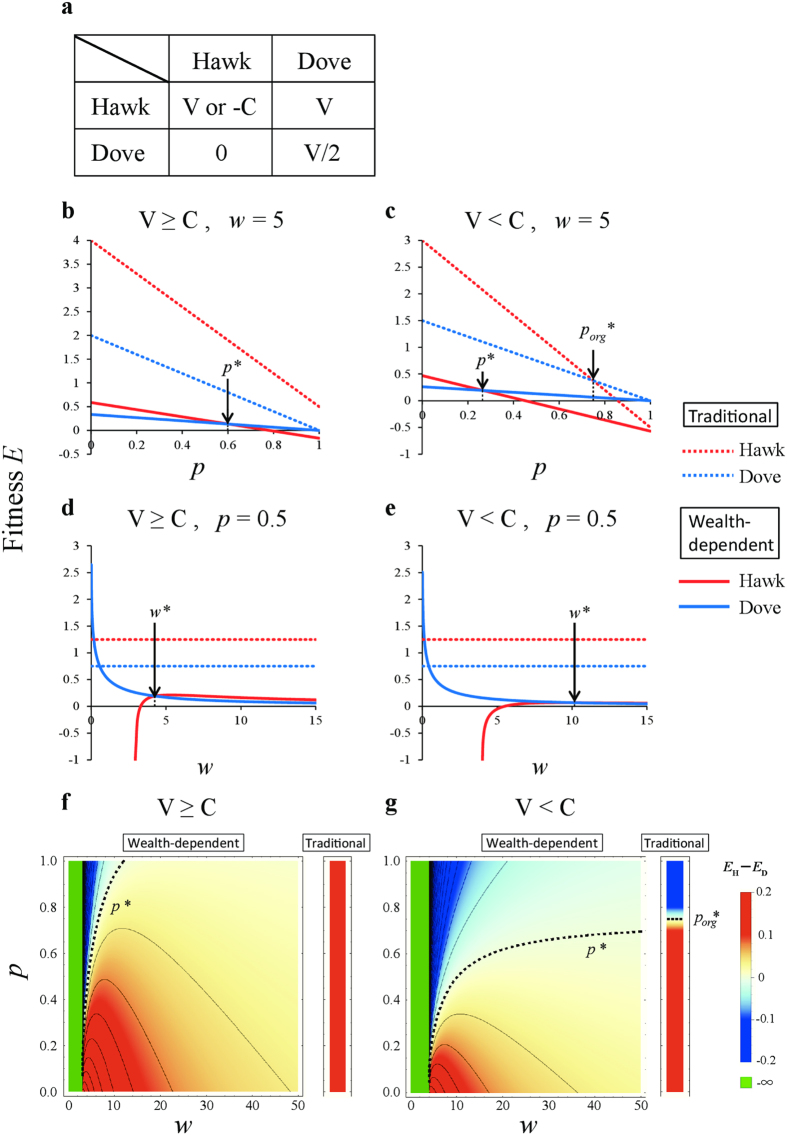
Wealth-dependent hawk-dove game. (**a**) Payoff matrix of the hawk-dove game. (**b**–**e**) The average payoffs of a hawk, *E*_H_(*u*), and a dove, *E*_D_(*u*), against *p* under (**b**) V ≥ C (*w* = 5, V = 4, C = 3), and (**c**) V < C (*w* = 5, V = 3, C = 4) and against *w* under (**d**) V ≥ C (*p* = 0.5, V = 4, C = 3) and (**e**) V < C (*p* = 0.5, V = 3, C = 4). The terms p*, *p*_org_*, and w* indicate that *E*_H_(*u*) = *E*_D_(*u*). For comparison, the payoffs of the hawk (red dashed line) and dove (blue dashed line) in the traditional game are included. (**f**–**g**) Phase diagram of current wealth, *w*, and the hawk frequency, *p*, when (**f**) V ≥ C (V = 4, C = 3) and (**g**) V < C (V = 3, C = 4). Outcomes (superiority) depend on the values of the differences in dynamic utility, *E*_H_(*u*) − *E*_D_(*u*): Dove (blue and green), hawk (red). The dashed line indicates equal utility, i.e., *E*_H_(*u*) = *E*_D_(*u*). The optimal strategy shifts from hawk to dove when a player becomes sufficiently poor. Note that payoffs are undefined in green areas.

**Figure 2:**
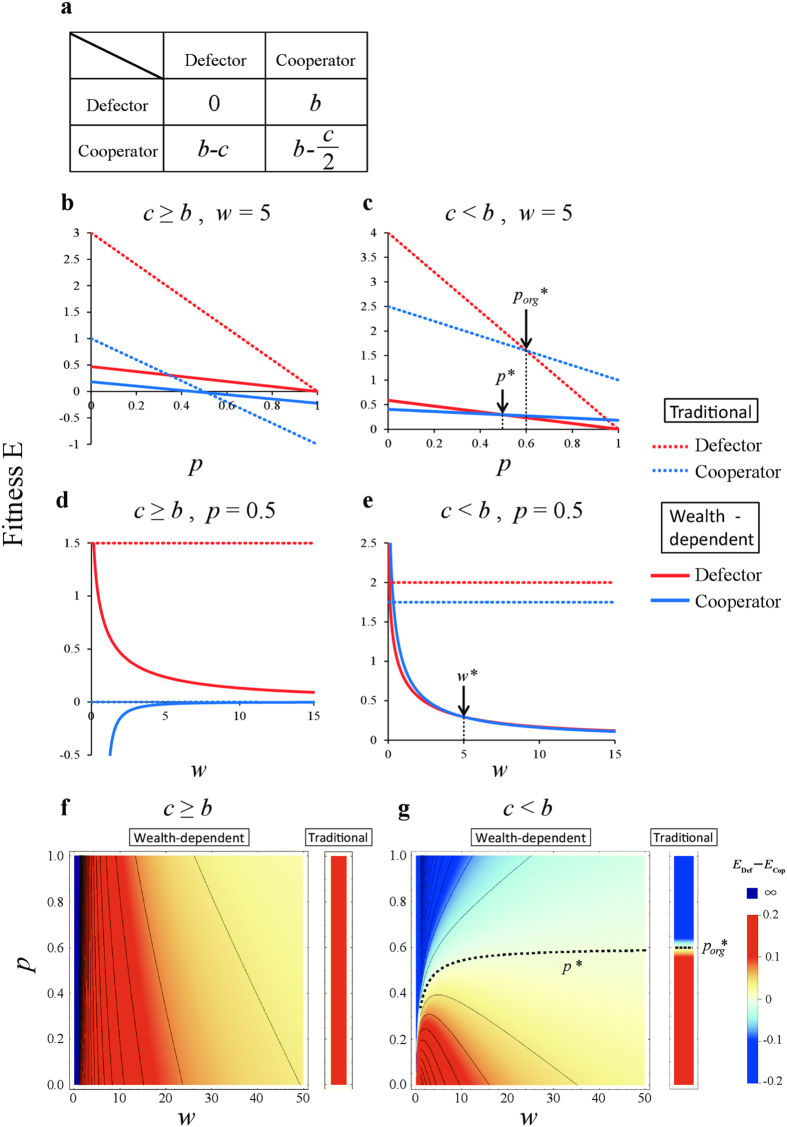
Wealth-dependent snowdrift game. (**a**) Payoff matrix of the snowdrift game. (**b**–**e**) The average payoffs of a defector, *E*_Def_(*u*), and a cooperator, *E*_Cop_(*u*), against *p* under (**b**) *c* ≥ *b* (*w* = 5, *c* = 4, *b* = 3), and (**c**) *c* < *b* (*w* = 5, *c* = 3, *b* = 4) and against *w* under (**d**) *c* ≥ *b* (*p* = 0.5, *c* = 4, *b* = 3), and (**e**) *c* < *b* (*p* = 0.5, *c* = 3, *b* = 4). The terms p*, *p*_org_*, and w* indicate that *E*_Def_(*u*) = *E*_Cop_(*u*). For comparison, the payoffs for the defector (red dashed line) and cooperator (blue dashed line) in the traditional game are included. (**f**–**g**) Phase diagram of current wealth, *w*, and the defector frequency, *p*, when (**f**) *c* ≥ *b* (*c* = 4, *b* = 3) and (**g**) *c* < *b* (*c* = 3, *b* = 4). Outcomes (superiority) depend on the difference in dynamic utility, *E*_Def_(*u*) − *E*_Cop_(*u*): Cooperator (blue), defector (red and purple). The dashed line indicates equal utility, i.e., *E*_Def_(*u*) = *E*_Cop_(*u*). If the cooperating cost, *c*, is smaller than the reward, *b*, the optimal strategy shifts from defection to cooperation when the player becomes sufficiently poor to ensure small positive gains. Note that payoffs are undefined in purple areas.

**Figure 3:**
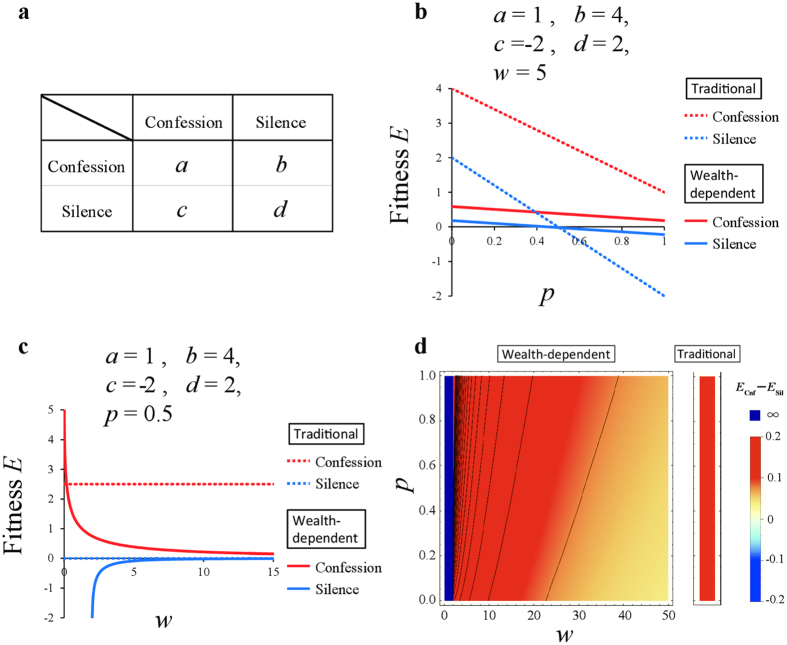
Wealth-dependent prisoner’s dilemma game. (**a**) Payoff matrix of the prisoner’s dilemma game. (**b** and **c**) Average payoffs of confession, *E*_Cnf_(*u*), and silence, *E*_Sil_(*u*), against *p* under (**b**) (*w* = 5, *a* = 1, *b* = 4, *c* = −2, *d* = 2) and against *w* under (**c**) (*p* = 0.5, *a* = 1, *b* = 4, *c* = −2, *d* = 2). For comparison, the payoffs of confession (red dashed line) and silence (blue dashed line) in the traditional game are included. (**d**) Phase diagram of current wealth, *w*, and the defector frequency, *p*, under *a* = 1, *b* = 4, *c* = −2 and *d* = 2. Outcomes (superiority) depend on the difference in dynamic utility *E*_Cnf_(*u*)–*E*_Sil_(*u*): Silence (blue: non-existent), confession (red and purple). In the prisoner’s dilemma game, confession is always superior to silence for all values of *p* and *w*. Note that the payoffs are undefined in purple areas.

Here, we introduce this state-dependent utility function 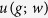 to game theory. Specifically, we re-evaluate the optimality of strategies in four traditional games—the hawk-dove game, the snowdrift game, the prisoner’s dilemma game and the stag hunt game^33^ —whose payoff matrices are shown in Fig. 1, 2, 3, 4. The first two games are types of chicken games.

## Models and Analyses

### The hawk-dove game

The payoff matrix of the hawk-dove game is given by two parameters: the victory reward, V, and fighting cost, C (Fig. 1a). Here, one hawk wins, and the other hawk loses in a contest between two hawks because the probability of their winning is equal (i.e., 0.5). Applying 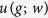 (Eq. (7)) to the hawk-dove game, the average payoffs of the hawk, *E*_H_, and the dove, *E*_D_, are calculated as follows:


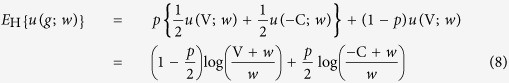



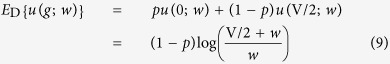


where *p* is the frequency of hawks, and *w* is the wealth of the player. As a numerical example, the average payoffs of *E*_H_ and *E*_D_ for *w* = 5 are plotted against the hawk frequency, *p*, when V ≥ C (Fig. 1b) and V < C (Fig. 1c). When V ≥ C, the optimal mix strategy (p* < 1) differs from the traditional hawk-dove game (*p*_org_* = 1) (Fig. 1b). When V < C, the proportion of doves becomes larger in the optimal mix strategy compared with the traditional hawk-dove game (p* < *p*_org_* < 1) (Fig. 1c). The average payoffs of *E*_H_ and *E*_D_ at *p* = 0.5 are also plotted against current wealth, *w*, when V ≥ C (Fig. 1d) and V < C (Fig. 1e). In both cases, the dove strategy becomes superior when *w* < w*, unlike the traditional hawk-dove game, in which the hawk is always superior to the dove. Note that w* is the amount of wealth when the average payoffs of the hawk and dove are equal (e.g., *E*_H_(*u*) = *E*_D_(*u*)) at *p* = 0.5 (Fig. 1d and e), where p*, *p*_org_*, and w* indicate that *E*_H_(*u*) = *E*_D_(*u*), respectably.

The phase diagrams are drawn for the hawk frequency, *p*, and current wealth, *w*, when V ≥ C (Fig. 1f) and V < C (Fig. 1g). Here, the dotted line p* = p*(V, C; *w*) shows the boundary where the utilities of the hawk and dove are equal, that is, *E*_H_ = *E*_D_:


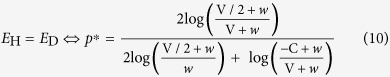


When V ≥ C, the dove strategy becomes superior to the hawk strategy if *w* ≤ C (=3), irrespective of *p*, or if *w* is slightly larger than C when *p* is close to one, i.e., *p* > p* (Fig. 1f). Thus, when V ≥ C, the optimal strategy is the dove if the player is very poor, even if the hawk was always optimal in the traditional game. The dove-superior region becomes much larger when V < C (Fig. 1f and g). This condition is satisfied if either *p* > p* or *w* < C (Fig. 1g). When V < C, the region where dove is optimal expands significantly when *w* decreases. Thus, in the hawk-dove game, the optimal strategy shifts from hawk to dove when the player becomes sufficiently poor (Fig. 1). Here, p* converges towards V/C when *w* increases towards ∞ (infinity):


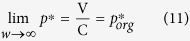


Interestingly the resulting value is equal to the equilibrium of the mixed evolutionarily stable strategy (ESS) in the traditional game, i.e., V/C = *p*_org_* (Eq. (11), see supplementary information).

### The snowdrift game

The payoff matrix of the snowdrift game is given by two parameters: the reward, *b*, and the cooperating (working) cost, *c* (Fig. 2a). Applying 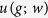 (Eq. (7)) to the snowdrift game, the average payoffs of the defector, *E*_Def_, and the cooperator, *E*_Cop_, are calculated as follows:


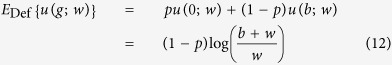



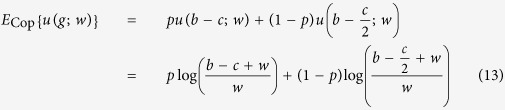


where *p* is the frequency of defectors. In the snowdrift game, the optimal strategy shifts from defection to cooperation when the player becomes sufficiently poor, and the cooperating cost, *c*, is smaller than the reward, *b* (Fig. 2). The average payoffs of *E*_Def_ and *E*_Cop_ for *w* = 5 were plotted against the defector frequency, *p*, when *c* ≥ *b* (Fig. 2b) and *c* < *b* (Fig. 2c). When *c* ≥ *b*, the optimal mix strategy (p* < 1) does not appear as in the traditional snowdrift game (*p*_org_* = 1) (Fig. 2b), nor does not appear as in the traditional snowdrift game when *c* < *b* (p* < *p*_org_* < 1) (Fig. 2c). The average payoffs of *E*_Def_ and *E*_Cop_ at *p* = 0.5 are plotted against current wealth, *w*, when *c* ≥ *b* (Fig. 2d) and *c* < *b* (Fig. 2e). Interestingly, when *c* > *b*, the results of a dynamic version are qualitatively the same as in the traditional snowdrift game. In the case of *c* < *b*, however, the defector strategy becomes superior when *w* < w* (Fig. 2d and e).

Phase diagrams were drawn for the defector frequency, *p*, and current wealth, *w*, when *c* ≥ *b* (Fig. 2f) and *c* < *b* (Fig. 2g). Here, the dotted line p* = p*(*b, c*; *w*) shows the boundary where the utility of the defector and co-operator are equal, that is, *E*_Def_ = *E*_Cop_:


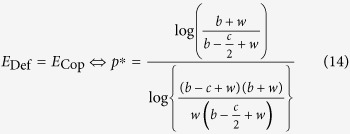


When *c* ≥ *b*, defecting is always superior to cooperation, even if the wealth of a player increases (Fig. 2f). Thus, qualitatively, the current dynamic version is same as in the traditional snowdrift game. In the case of *c* < *b*, however, unlike the traditional snowdrift game, in which defector is always superior to cooperator (Fig. 2d and f), the cooperator becomes superior if *w* < w* (Fig. 2e and g). Specifically, the region where cooperation is optimal expands significantly when *w* decreases (Fig. 2g). Notably, p* is undefined if *w* ≤ *c* − *b*, where *E*_Def_ ≫ *E*_Cop_, indicating that defection is always advantageous (purple area in Fig. 2g).

Here, p* converges to *c*/(2*b* − *c*) when *w* increases towards ∞:


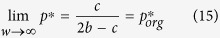


where this convergent value is equal to the equilibrium of the mixed ESS in the traditional snowdrift game, i.e., *c*/(2*b* − *c*) = *p*_org_* (Eq. (15), see Supplementary Information).

### The prisoner’s dilemma game

The payoff matrix of the prisoner’s dilemma game is given by four parameters *a, b, c* and *d*, where *b* > *d* > *a* > *c* and *d* > (*b* + *c*)/2 (Fig. 3a). Applying 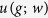 (Eq. (7)) to the prisoner’s dilemma game, the average payoffs of confession, *E*_Cnf_, and silence, *E*_Sil_, are as follows:


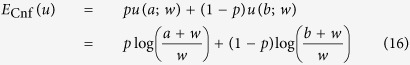



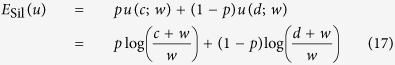


where *p* is the frequency of confession. We plotted the average payoffs of *E*_Cnf_ and *E*_Sil_ for *w* = 5 against the confession frequency, *p*, when *a* = 1, *b* = 4, *c* = −2, *d* = 2 (Fig. 3b). The optimal mixed strategy does not appear in this game, similar to the traditional prisoner’s dilemma game (Fig. 3b and c). Here, we can calculate p* = p*(*a, b, c, d*; *w*), that is, *E*_Cnf_ = *E*_Sil_:


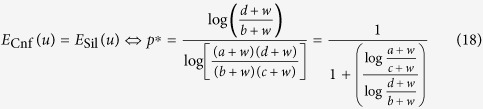


Equation (18) is always larger than 1 or smaller than 0, i.e., p* > 1 or p* < 0. In the prisoner’s dilemma game, confession is always superior to silence for any wealth, *w* (Fig. 3d). Here, p* converges to the same value (*p*_org_* > 1 or *p*_org_* < 0) as in the traditional game when *w* approaches ∞:


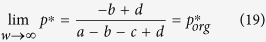


Thus the convergent value becomes equal to the equilibrium of the traditional game (Eq. (19), see Supplementary Information). Numerically we found p* > *p*_org_* > 1 or 0 > p* > *p*_org_*. However, we could not prove these inequalities of *p*_org_* and p* analytically. Notably, p* is undefined if *w* ≤ −*c*, where *E*_Cnf_ ≫ *E*_Sil_, indicating that defecting is always advantageous (purple area in Fig. 3d).

### Stag hunt game

**Figure 4:**
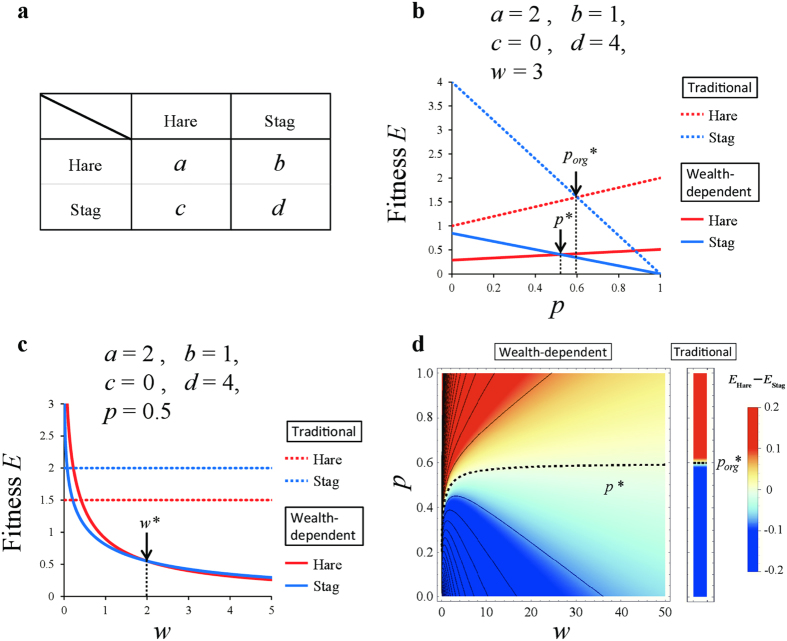
Wealth-dependent stag hunt game. (**a**) Payoff matrix of the stag hunt game. (**b** and **c**) Average payoffs of the hare, *E*_Hare_(*u*), and stag, *E*_Stag_(*u*), against *p* under (**b**) (*w* = 5, *a* = 2, *b* = 1, *c* = 0, *d* = 4) and against *w* under (**c**) (*p* = 0.5, *a* = 2, *b* = 1, *c* = 0, *d* = 4). For comparison, the payoffs of the hare (red dashed line) and stag (blue dashed line) in the traditional game are included. (**d**) Phase diagram of current wealth, *w*, and the hare frequency, *p*, under *a* = 2, *b* = 1, *c* = 0 and *d* = 4. Outcomes (superiority) depend on the difference in dynamic utility *E*_Hare_(*u*) – *E*_Stag_(*u*): Stag (blue), hare (red). A poor player chooses the sure gain of a small hare instead of an uncertain large stag.

The payoff matrix of the stag hunt game is given by four parameters *a, b, c* and *d*, where *d* > *a* ≥ *c* > *b* (Fig. 4a). Applying 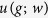 (Eq. (7)) to the stag hunt game, the average payoffs of the hare, *E*_Hare_, and the stag, *E*_Stag_, are as follows:


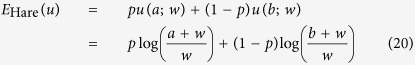



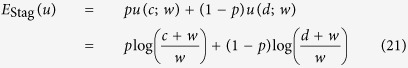


where *p* is the frequency of hares. We plotted the average payoffs of *E*_Hare_ and *E*_Stag_ for *w* = 3 against hare frequency, *p*, when *a* = 2, *b* = 1, *c* = 0, and *d* = 4 (Fig. 4b). In this case, the optimal mix strategy appears as in both the wealth-dependent and traditional stag hunt games (p* < *p*_org_* < 1). The average payoffs of *E*_Hare_ and *E*_Stag_ at *p* = 0.6 are plotted against current wealth, *w* (Fig. 4c). Here, we can calculate p* = p*(*a, b, c, d*; *w*), that is, *E*_Hare_ = *E*_Stag_:


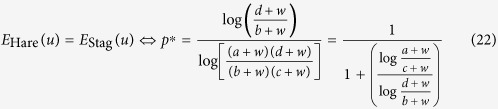


Equation (22) is always larger than 0 and smaller than 1, i.e., 0 < p* < 1. In the dynamic version of the stag hunt game, the optimal strategy shifts from stag to hare when the player becomes sufficiently poor (Fig. 4d). Here, p* converges to the same value as the stable mixed strategy ESS in the traditional stag hunt game when *w* approaches ∞:


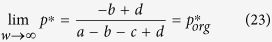


Interestingly the resulting value is equal to the equilibrium of the changing point of optimal strategy in the traditional game, i.e., (−*b* + *d*)/(*a* − *b* − *c*+*d*) = *p*_org_* (Eq. (23), see supplementary information). Numerically we found *p*_org_* > p*, as in Hawk-Dove game and snowdrift game. However, we could not prove these inequalities of *p*_org_* and p* analytically.

## Discussion

Our analyses showed that in all four games analyzed, the traditional game is equivalent to the current dynamic game when the wealth of the player is infinite, suggesting that optimal strategies may be different under limited wealth. In the hawk-dove game, snowdrift game and stag hunt game, the optimal strategy becomes different from the traditional solution. However, the prisoner’s dilemma game demonstrates that a dynamic version of game theory can be quite similar to traditional game theory in some situations. Therefore, we must verify when the dynamic version departs radically from traditional game theory.

Individuals change their behavioral patterns depending on their current status. For example, among human beings, village people living in harsh environments (e.g., deserts or polar regions) frequently face survival crises that affect the entire village. They survive by building closely collaborative relationships, helping one another and sharing small amounts of leftover food^34^,^35^. In some cases, these communities develop their own customs (e.g., precepts) and promote cooperation with social penalties^36^. In contrast, the wealthiest 1% controls half of the global wealth by seeking short-term profits via huge asset utilization^37^,^38^,^39^.

The results of wealth-dependent games may represent the nature of our human society. In dynamic hawk-dove games, when a player becomes sufficiently poor, the optimal strategy shifts from hawk to dove to avoid the fighting cost, C (Fig. 1). In dynamic snowdrift games, if the cooperating cost, *c*, is smaller than the reward, *b*, then the optimal strategy shifts from defection to cooperation when the player becomes sufficiently poor to ensure positive gains in any outcome (Fig. 2). In the stag hunt game, a poor player chooses the sure gain of a small hare, instead of an uncertain large stag (Fig. 4). Note that this choice for smaller certain gains is expressed only by poor people^31^. It is known as the sure-thing principle^40^, also shown in the Allais Paradox^31^,^41^.

The sure-thing principle can be illustrated with the following example. Consider a game in which players have two choices. For the first choice, the payoff is 3 with a probability of *p* = 1.0 (100%), while for the second choice, it is either 8 with *p* = 0.5 or 0 with *p* = 0.5. When the state of the player (current wealth) is 1, the utility of the first and second choices (*E*_1_{*u*} and *E*_2_{*u*}) is as follows:

(1) First choice:


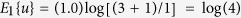


(2) Second choice:


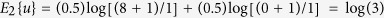


This indicates that the player chooses the first choice. This situation, involving a small sure return, may appear in many chicken games, as in the current three games. However, if this game is repeated, then the current wealth of the player should accumulate, and the second choice becomes superior soon thereafter. The critical level of wealth is calculated from *E*_1_{*u*} = *E*_2_{*u*}: (1.0)log[(3 + *w*)/*w*] =  (0.5)log[(8 + *w*)/*w*], and we obtain *w* = 9/2. As shown in this example, when the player becomes sufficiently wealthy, the optimal strategy approaches a condition that ensures the maximum arithmetic gain.

Our results may imply that the conditions for cooperation are much broader than expected in traditional game theory. We show that poor players exhibit cooperation in three types of chicken games, but not in the prisoner’s dilemma game. However, we are unsure whether this exhibition of cooperation by the poor players occurs in other chicken games or any other games. Many attempts have been made to explain the evolution of cooperation by introducing additional complexity^10^,^11^,^12^,^13^,^14^,^15^,^16^,^17^,^18^,^19^,^20^,^21^,^22^,^23^,^24^,^25^,^26^. Here, we find a new condition for the evolution of cooperation by incorporating dynamic decision making.

Note that the current dynamic utility was developed for the behavioral dynamics of an individual in the fields of behavioral ecology, microeconomics and operations research. This method can be applicable to evolutionary games such as ESS analysis. However, the current wealth of a player should be considered in the optimality analyses. Therefore, each value (numbers) in a payoff matrix should be treated as a real amount of gain, instead of a utility^28^. Our analyses of behavioral dynamics are also different from evolutionary adaptation in a stochastic environment, where geometric mean fitness is often used^32^. Even though the analyses of wealth dynamics (of an individual) are mathematically quite similar to geometric mean analyses of population growth under a stochastic environment, the former type of analysis involves the optimization of dynamic behavior at the individual level, while the latter involves the optimization of population size over generations. Therefore, we should not confuse the current individual analyses with the geometric mean fitness in evolutionary ecology.

We should also note that the current analyses are all based on one-trial games. Here, we cannot discuss stable equilibria. Nash equilibrium cannot be easily calculated in dynamic games, such as repeated and evolutionary games, because the optimal solutions change dynamically along with temporal changes in the current wealth of players. The concept of Nash equilibrium seems to be correct, but there are no simple solutions available for stable equilibrium in dynamic games. However, the dynamically optimal solutions for various chicken games deviate from the traditional solutions toward cooperation. In other words, poor players tend to behave more cooperatively. Therefore, cooperative behavior should be more common than previously expected.

## Additional Information

**How to cite this article**: Ito, H. *et al*. The promotion of cooperation by the poor in dynamic chicken games. *Sci. Rep.* **7**, 43377; doi: 10.1038/srep43377 (2017).

**Publisher's note:** Springer Nature remains neutral with regard to jurisdictional claims in published maps and institutional affiliations.

## Supplementary Material

Supplementary Information
