# Supplementary material for: The promotion of cooperation by the poor in dynamic chicken games
Source: Sci Rep. 2017 Feb 24;7:43377. doi: 10.1038/srep43377 (PMC5324166; doi:10.1038/srep43377)
Supplement: Supplementary Information [file srep43377-s1.pdf]

Supplementary Information

The promotion of cooperation by the poor in dynamic chicken games

Hiromu Ito · Yuki Katsumata · Eisuke Hasegawa · Jin Yoshimura

Supporting Information

Derivation of the convergence value of each p^*

1. Derivation of Equation (11) for the hawk-dove game

Here, we calculate the convergence value of p^* (Equation (10)):

$$p^* = \frac{2\log\left(\frac{V/2+w}{V+w}\right)}{2\log\left(\frac{V/2+w}{w}\right)+\log\left(\frac{-C+w}{V+w}\right)} \qquad (10)$$

Equation (10) is rewritten as follows:

$$p^* = \frac{2\log\left(\frac{V/2+w}{w}\right)-2\log\left(\frac{V+w}{w}\right)}{2\log\left(\frac{V/2+w}{w}\right)+\log\left(\frac{-C+w}{w}\right)-\log\left(\frac{V+w}{w}\right)}$$

$$= \frac{2\log\left(\frac{V/2}{w}+1\right)-2\log\left(\frac{V}{w}+1\right)}{2\log\left(\frac{V/2}{w}+1\right)+\log\left(\frac{-C}{w}+1\right)-\log\left(\frac{V}{w}+1\right)} \qquad (S1)$$

Calculate the limit of p^* (Equation (S1)) as w approaches infinity:

$$\lim_{w\to\infty} p^* = \lim_{w\to\infty}\frac{2\log\left(\frac{V/2}{w}+1\right)-2\log\left(\frac{V}{w}+1\right)}{2\log\left(\frac{V/2}{w}+1\right)+\log\left(\frac{-C}{w}+1\right)-\log\left(\frac{V}{w}+1\right)}$$

$$= \frac{2\log(0+1)-2\log(0+1)}{2\log(0+1)+\log(0+1)-\log(0+1)} = \frac{0}{0} \qquad (S2)$$

The answer to Equation (S2) is an indeterminate form. Therefore, we apply l'Hôpital's rule to Equation (S1):

$$\lim_{w\to\infty} p^* = \lim_{w\to\infty} \frac{2\log\left(\frac{V/2+w}{w}\right) - 2\log\left(\frac{V+w}{w}\right)}{2\log\left(\frac{V/2+w}{w}\right) + \log\left(\frac{-C+w}{w}\right) - \log\left(\frac{V+w}{w}\right)}$$

$$= \lim_{w\to\infty} \frac{\frac{d}{dw}\left\{2\log\left(\frac{V/2+w}{w}\right) - 2\log\left(\frac{V+w}{w}\right)\right\}}{\frac{d}{dw}\left\{2\log\left(\frac{V/2+w}{w}\right) + \log\left(\frac{-C+w}{w}\right) - \log\left(\frac{V+w}{w}\right)\right\}}$$

$$= \lim_{w\to\infty} \frac{\frac{-V}{V/2+w} + \frac{2V}{V+w}}{\frac{-V}{V/2+w} + \frac{C}{-C+w} + \frac{V}{V+w}} \qquad \text{(S3)}$$

Multiply both the denominator and numerator of Equation (S3) by (V/2+w)^2 (V+w)^2 (-C+w):

$$\lim_{w\to\infty} p^* = \lim_{w\to\infty} \frac{\left(\frac{V}{2}+w\right)^2 (V+w)^2(-C+w)\left(\frac{-V}{V/2+w} + \frac{2V}{V+w}\right)}{\left(\frac{V}{2}+w\right)^2 (V+w)^2(-C+w)\left(\frac{-V}{V/2+w} + \frac{C}{-C+w} + \frac{V}{V+w}\right)}$$

$$= \lim_{w\to\infty} \frac{-V\left(\frac{V}{2}+w\right)(V+w)^2(-C+w) + 2V\left(\frac{V}{2}+w\right)^2(V+w)(-C+w)}{-V\left(\frac{V}{2}+w\right)(V+w)^2(-C+w) + C\left(\frac{V}{2}+w\right)^2(V+w)^2 + V\left(\frac{V}{2}+w\right)^2(V+w)(-C+w)} \qquad \text{(S4)}$$

Divide both the denominator and numerator of Equation (S4) by w^4:

$$\lim_{w\to\infty} p^*$$

$$= \lim_{w\to\infty} \frac{-V\left(\frac{V}{2w}+1\right)\left(\frac{V}{w}+1\right)^2\left(\frac{-C}{w}+1\right) + 2V\left(\frac{V}{2w}+1\right)^2\left(\frac{V}{w}+1\right)\left(\frac{-C}{w}+1\right)}{-V\left(\frac{V}{2w}+1\right)\left(\frac{V}{w}+1\right)^2\left(\frac{-C}{w}+1\right) + C\left(\frac{V}{2w}+1\right)^2\left(\frac{V}{w}+1\right)^2 + V\left(\frac{V}{2w}+1\right)^2\left(\frac{V}{w}+1\right)\left(\frac{-C}{w}+1\right)}$$

$$= \frac{-V+2V}{-V+C+V}$$

$$= \frac{V}{C} = p^*_{org} \qquad \text{(S5)}$$

2. The snowdrift game (Eq. (15))

Here, we calculate the convergence value of p^* (Equation (14)):

$$p^* = \frac{\log\left(\frac{b+w}{b-\frac{c}{2}+w}\right)}{\log\left\{\frac{(b-c+w)(b+w)}{w(b-\frac{c}{2}+w)}\right\}} \qquad (14)$$

Equation (14) is rewritten as follows:

$$p^* = \frac{\log\left(\frac{b+w}{w}\right) - \log\left(\frac{b-\frac{c}{2}+w}{w}\right)}{\log\left(\frac{b-c+w}{w}\right) + \log\left(\frac{b+w}{w}\right) - \log\left(\frac{b-\frac{c}{2}+w}{w}\right)}$$

$$= \frac{\log\left(\frac{b}{w}+1\right) - \log\left(\frac{b-\frac{c}{2}}{w}+1\right)}{\log\left(\frac{b-c}{w}+1\right) + \log\left(\frac{b}{w}+1\right) - \log\left(\frac{b-\frac{c}{2}}{w}+1\right)} \qquad \text{(S6)}$$

Calculate the limit of $p*$ (Equation (S6)) as w approaches infinity:

$$\lim_{w\to\infty} p^* = \lim_{w\to\infty} \frac{\log\left(\frac{b}{w}+1\right) - \log\left(\frac{b-\frac{c}{2}}{w}+1\right)}{\log\left(\frac{b-c}{w}+1\right) + \log\left(\frac{b}{w}+1\right) - \log\left(\frac{b-\frac{c}{2}}{w}+1\right)}$$

$$= \frac{\log(0+1) - \log(0+1)}{\log(0+1) + \log(0+1) - \log(0+1)} = \frac{0}{0} \qquad \text{(S7)}$$

The answer to Equation (S7) is an indeterminate form. Therefore, we apply l'Hôpital's rule to Equation (S6):

$$\lim_{w\to\infty} p^* = \lim_{w\to\infty} \frac{\log\left(\frac{b+w}{w}\right) - \log\left(\frac{b-\frac{c}{2}+w}{w}\right)}{\log\left(\frac{b-c+w}{w}\right) + \log\left(\frac{b+w}{w}\right) - \log\left(\frac{b-\frac{c}{2}+w}{w}\right)}$$

$$= \lim_{w\to\infty} \frac{\frac{d}{dw}\left\{\log\left(\frac{b+w}{w}\right) - \log\left(\frac{b-\frac{c}{2}+w}{w}\right)\right\}}{\frac{d}{dw}\left\{\log\left(\frac{b-c+w}{w}\right) + \log\left(\frac{b+w}{w}\right) - \log\left(\frac{b-\frac{c}{2}+w}{w}\right)\right\}}$$

$$= \lim_{w\to\infty} \frac{\frac{-b}{b+w} + \frac{b-\frac{c}{2}}{b-\frac{c}{2}+w}}{\frac{-b+c}{b-c+w} - \frac{b}{b+w} + \frac{b-\frac{c}{2}}{b-\frac{c}{2}+w}} \qquad \text{(S8)}$$

Multiply both the denominator and numerator of Equation (S8) by $(b+w)^2$ $(b\text{-}c/2+w)^2$ $(b\text{-}c+w)$:

$$\lim_{w\to\infty} p^* = \lim_{w\to\infty} \frac{(b+w)^2\left(b-\frac{c}{2}+w\right)^2(b-c+w)\left(\frac{-b}{b+w} + \frac{b-\frac{c}{2}}{b-\frac{c}{2}+w}\right)}{(b+w)^2\left(b-\frac{c}{2}+w\right)^2(b-c+w)\left(\frac{-b+c}{b-c+w} - \frac{b}{b+w} + \frac{b-\frac{c}{2}}{b-\frac{c}{2}+w}\right)}$$

$$= \lim_{w\to\infty} \frac{-b(b+w)\left(b-\frac{c}{2}+w\right)^2(b-c+w) + (b-\frac{c}{2})(b+w)^2\left(b-\frac{c}{2}+w\right)(b-c+w)}{(-b+c)(b+w)^2\left(b-\frac{c}{2}+w\right)^2 - b(b+w)\left(b-\frac{c}{2}+w\right)^2(b-c+w) + (b-\frac{c}{2})(b+w)^2\left(b-\frac{c}{2}+w\right)(b-c+w)} \qquad \text{(S9)}$$

Divide both the denominator and numerator of Equation (S9) by w^4:

$$\lim_{w\to\infty} p^*$$

$$= \lim_{w\to\infty} \frac{-b\left(\frac{b}{w}+1\right)\left(\frac{b-\frac{c}{2}}{w}+1\right)^2\left(\frac{b-c}{w}+1\right) + (b-\frac{c}{2})\left(\frac{b}{w}+1\right)^2\left(\frac{b-\frac{c}{2}}{w}+1\right)\left(\frac{b-c}{w}+1\right)}{(-b+c)\left(\frac{b}{w}+1\right)^2\left(\frac{b-\frac{c}{2}}{w}+1\right)^2 - b\left(\frac{b}{w}+1\right)\left(\frac{b-\frac{c}{2}}{w}+1\right)^2\left(\frac{b-c}{w}+1\right) + (b-\frac{c}{2})\left(\frac{b}{w}+1\right)^2\left(\frac{b-\frac{c}{2}}{w}+1\right)\left(\frac{b-c}{w}+1\right)}$$

$$= \frac{-b+b-\frac{c}{2}}{-b+c-b+b-\frac{c}{2}}$$

$$= \frac{c}{2b-c} = p^*_{org} \qquad \text{(S10)}$$

3. The prisoner's dilemma and stag hunt games (Equations (19) and (23))

Here, we calculate the convergence value of $p*$ (Equations (18) and (22)):

$$p^* = \frac{\log\left(\frac{d+w}{b+w}\right)}{\log\left[\frac{(a+w)(d+w)}{(b+w)(c+w)}\right]} \qquad \text{(18) and (22)}$$

Equations (18) and (22) are rewritten as follows:

$$p^* = \frac{\log\left(\frac{d+w}{w}\right) - \log\left(\frac{b+w}{w}\right)}{\log\left(\frac{a+w}{w}\right) - \log\left(\frac{b+w}{w}\right) - \log\left(\frac{c+w}{w}\right) + \log\left(\frac{d+w}{w}\right)}$$

$$= \frac{\log\left(\frac{d}{w}+1\right) - \log\left(\frac{b}{w}+1\right)}{\log\left(\frac{a}{w}+1\right) - \log\left(\frac{b}{w}+1\right) - \log\left(\frac{c}{w}+1\right) + \log\left(\frac{d}{w}+1\right)} \qquad \text{(S11)}$$

Calculate the limit of $p*$ (Equation (S11)) as w approaches infinity:

$$\lim_{w\to\infty} p^* = \lim_{w\to\infty} \frac{\log\left(\frac{d}{w}+1\right) - \log\left(\frac{b}{w}+1\right)}{\log\left(\frac{a}{w}+1\right) - \log\left(\frac{b}{w}+1\right) - \log\left(\frac{c}{w}+1\right) + \log\left(\frac{d}{w}+1\right)}$$

$$= \frac{\log(0+1) - \log(0+1)}{\log(0+1) - \log(0+1) - \log(0+1) + \log(0+1)} = \frac{0}{0} \qquad \text{(S12)}$$

The answer to Equation (S12) is an indeterminate form. Therefore, we apply l'Hôpital's rule to Equation (S11):

$$\lim_{w\to\infty} p^* = \lim_{w\to\infty} \frac{\log\left(\frac{d+w}{w}\right) - \log\left(\frac{b+w}{w}\right)}{\log\left(\frac{a+w}{w}\right) - \log\left(\frac{b+w}{w}\right) - \log\left(\frac{c+w}{w}\right) + \log\left(\frac{d+w}{w}\right)}$$

$$= \lim_{w\to\infty} \frac{\frac{d}{dw}\left\{\log\left(\frac{d+w}{w}\right) - \log\left(\frac{b+w}{w}\right)\right\}}{\frac{d}{dw}\left\{\log\left(\frac{a+w}{w}\right) - \log\left(\frac{b+w}{w}\right) - \log\left(\frac{c+w}{w}\right) + \log\left(\frac{d+w}{w}\right)\right\}}$$

$$= \lim_{w \to \infty} \frac{\frac{-d}{d+w} + \frac{b}{b+w}}{\frac{-a}{a+w} + \frac{b}{b+w} + \frac{c}{c+w} - \frac{d}{d+w}} \qquad \text{(S13)}$$

Multiply both the denominator and numerator of Equation (S13) by (a+w)(b+w)(c+w)(d+w):

$$\lim_{w \to \infty} p^* = \lim_{w \to \infty} \frac{(a+w)(b+w)(c+w)(d+w)\left(\frac{-d}{d+w} + \frac{b}{b+w}\right)}{(a+w)(b+w)(c+w)(d+w)\left(\frac{-a}{a+w} + \frac{b}{b+w} + \frac{c}{c+w} - \frac{d}{d+w}\right)}$$

$$= \lim_{w \to \infty} \frac{-d(a+w)(b+w)(c+w) + b(a+w)(c+w)(d+w)}{-a(b+w)(c+w)(d+w) + b(a+w)(c+w)(d+w) + c(a+w)(b+w)(d+w) - d(a+w)(b+w)(c+w)} \qquad \text{(S14)}$$

Divide both the denominator and numerator of Equation (S14) by w^3:

$$\lim_{w \to \infty} p^*$$

$$= \lim_{w \to \infty} \frac{-d\left(\frac{a}{w}+1\right)\left(\frac{b}{w}+1\right)\left(\frac{c}{w}+1\right) + b\left(\frac{a}{w}+1\right)\left(\frac{c}{w}+1\right)\left(\frac{d}{w}+1\right)}{-a\left(\frac{b}{w}+1\right)\left(\frac{c}{w}+1\right)\left(\frac{d}{w}+1\right) + b\left(\frac{a}{w}+1\right)\left(\frac{c}{w}+1\right)\left(\frac{d}{w}+1\right) + c\left(\frac{a}{w}+1\right)\left(\frac{b}{w}+1\right)\left(\frac{d}{w}+1\right) - d\left(\frac{a}{w}+1\right)\left(\frac{b}{w}+1\right)\left(\frac{c}{w}+1\right)}$$

$$= \frac{-d+b}{-a+b+c-d}$$

$$= \frac{-b+d}{a-b-c+d} = p^*_{org} \qquad \text{(S15)}$$
